# Supplementary material for: Antibiotic Tolerance Indicative of Persistence Is Pervasive among Clinical Streptococcus pneumoniae Isolates and Shows Strong Condition Dependence
Source: Microbiol Spectr. 2022 Nov 14;10(6):e02701-22. doi: 10.1128/spectrum.02701-22 (PMC9769776; doi:10.1128/spectrum.02701-22)
Supplement: Supplemental file 1 — Supplemental material. Download spectrum.02701-22-s0001.pdf, PDF file, 0.7 MB [file spectrum.02701-22-s0001.pdf]

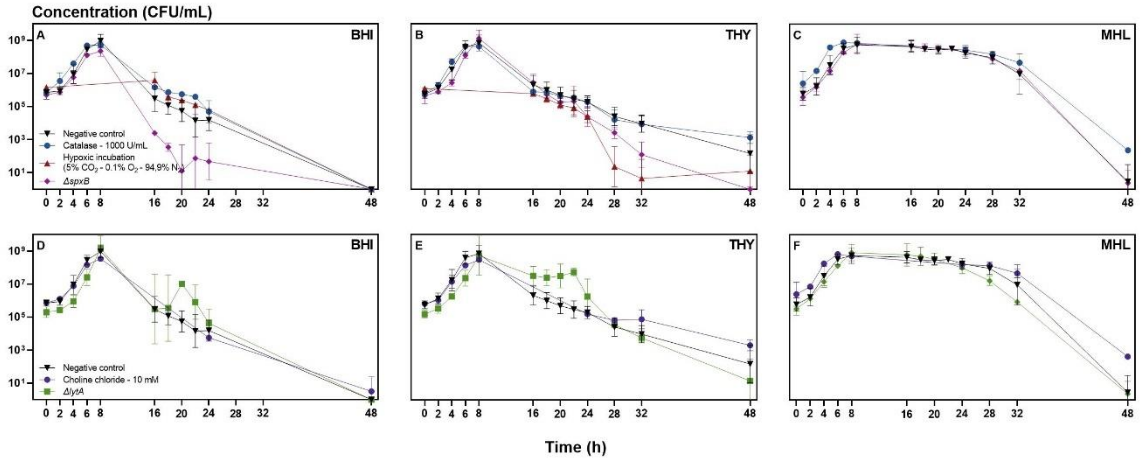

Figure S1: **The self-limiting *in vitro* nature of *S. pneumoniae* is not counteracted in BHI and THY by different strategies adopted to avoid H_2O_2 or autolysis.**

We compared the effect of different strategies to counteract the effects of pyruvate oxidase (A, B and C) or autolysin (D, E and F) in planktonic growth curves of *S. pneumoniae* D39 in BHI (Brain Heart Infusion broth, A and D), THY (Todd-Hewitt broth supplemented with 0.5% Yeast extract, B and E) and MHL (Mueller-Hinton broth supplemented with 5% Lysed horse blood, C and F). The strong reduction of viable bacteria after 8 hours of growth is still observed despite the adopted strategies in the media BHI and THY, but MHL abolishes the self-limiting *in vitro* nature of *S. pneumoniae*. The experiments were performed in duplicates or triplicates and each value is presented as the mean ± standard deviation (n ≥ 2).

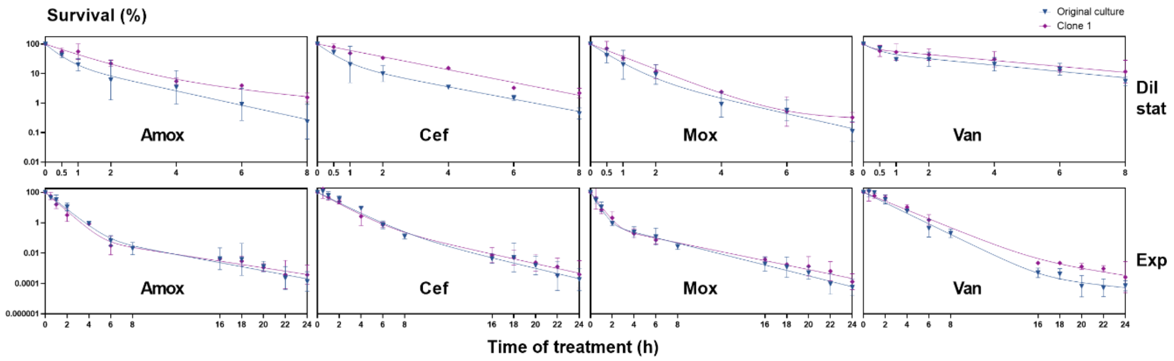

Figure S2: **The antibiotic tolerance of surviving *S. pneumoniae* cells is transient and non-deterministically inherited by daughter cells.**
Fitting of a non-linear fixed-effect model to log-transformed kill curves of amoxicillin (amox), cefuroxime (cef), moxifloxacin (mox) and vancomycin (van) against *S. pneumoniae* D39 planktonic bacteria. AB-tolerant *S. pneumoniae* D39 clones were recovered after 6 (Dil stat) or 18 (Exp) hours of treatment during the initial time-kill assay, regrown without antibiotic and preserved at -80°C. For one of these clones arising from potential persister cells, survival was determined over 8 or 24 hours of antibiotic treatment with amoxicillin (amox), cefuroxime (cef), moxifloxacin (mox) and vancomycin (van) in the diluted stationary (Dil stat) or the exponential growth phase (Exp). Killing dynamic patterns of the randomly selected clones were similar to the original culture (two-way ANOVA). The experiments were performed in triplicates and each value is presented as the mean ± standard deviation (n = 3).

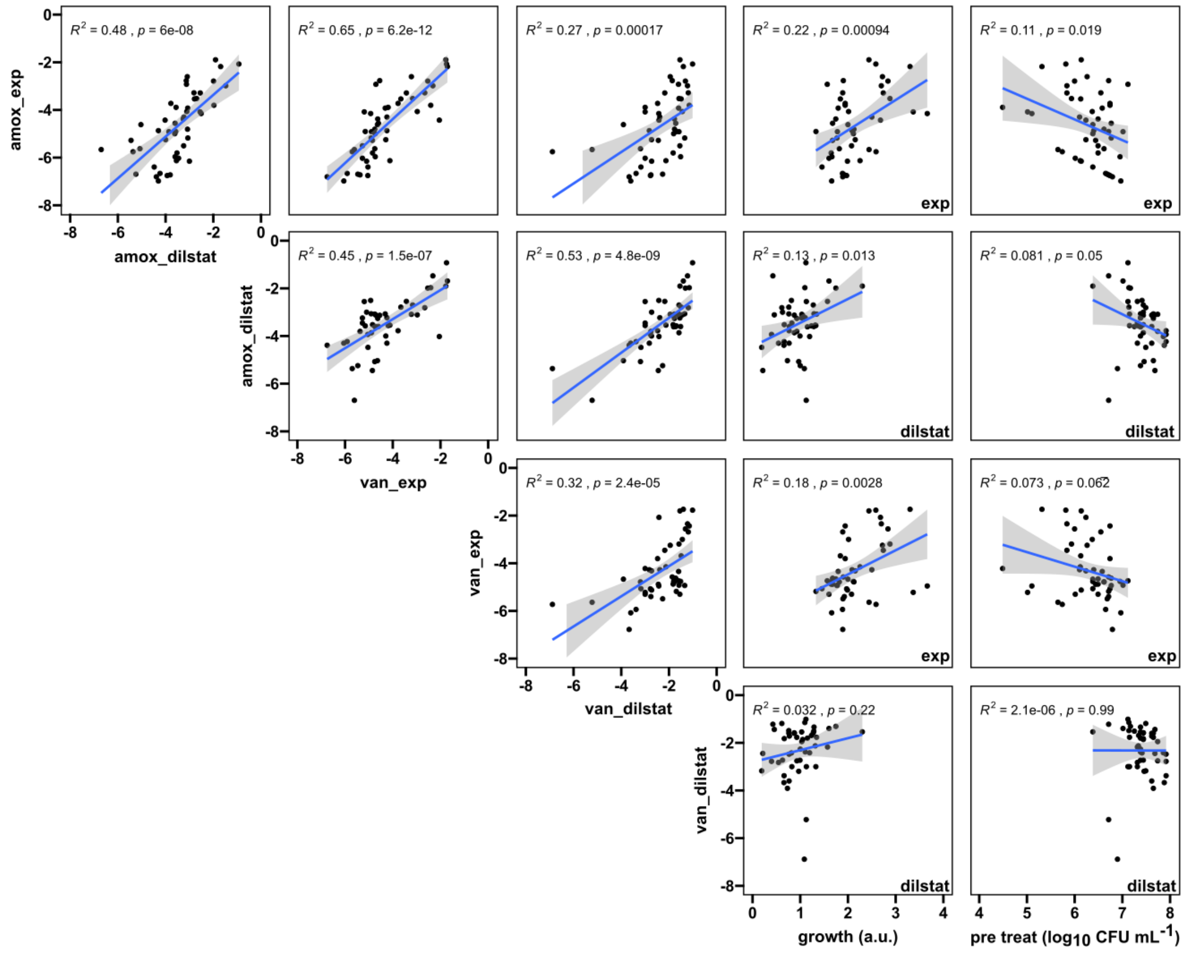

Figure S3: **Correlation analysis of survival fractions between different conditions show strong correlations between antibiotics (amoxicillin and vancomycin) and growth phases (diluted stationary and exponential growth phase).**

Individual correlations between the survival rates in 4 different conditions: treatment with amoxicillin (amox) or vancomycin (van) in the diluted stationary (dilstat) or exponential (exp) growth phase. In addition, the correlation with the corresponding control (growth in absence of antibiotics during the period of treatment in exponential or diluted stationary phase conditions) and with the initial CFU before treatment (pre treat) is given. Pearson correlation coefficients (R^2) are given for each correlation. A.u., arbitrary units.

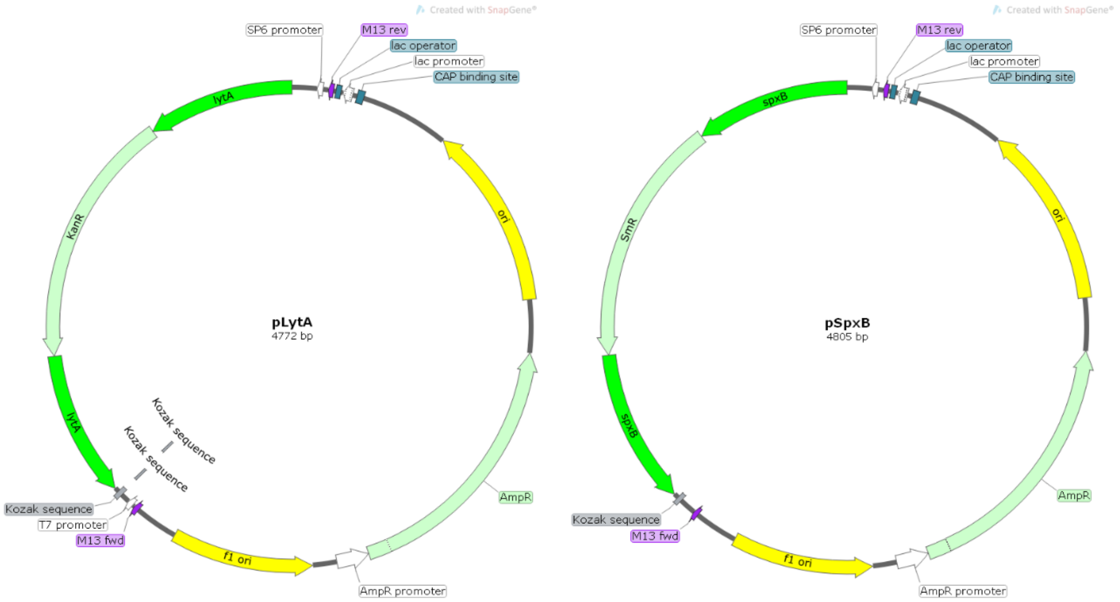

Figure S4: **Schematic overview of the constructed plasmids to generate knockout mutants of *lytA* (pLytA) and *spxB* (pSpxB) in *S. pneumoniae* D39.**

The plasmid contains the first and last 500 bp of the gene (*lytA* or *spxB*) disrupted by an antibiotic resistance marker (kanamycin cassette for *lytA* and streptomycin cassette for *spxB*).

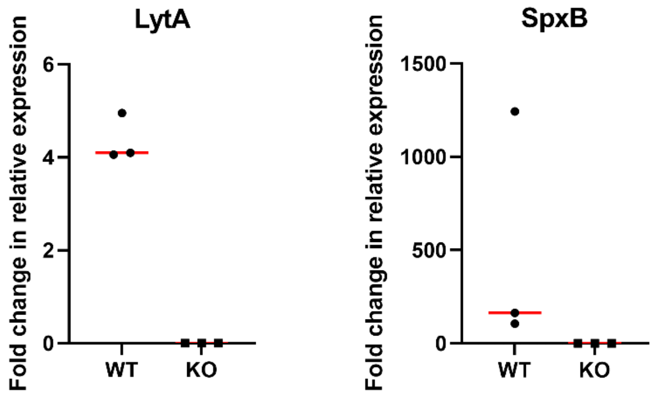

Figure S5: **The knockout mutants of the enzymes autolysin (LytA) and pyruvate oxidase (SpxB) show no mRNA expression.**

The data represent the number of fold-changes in mRNA levels of knockout mutants (KO) and wild-types (WT). The mean is given as the red line with the individual datapoints as dots (n = 1x3).

Table S1: ***S. pneumoniae* D39 is susceptible to the antibiotics amoxicillin, cefuroxime, moxifloxacin and vancomycin according to the EUCAST breaking points. Minimum inhibitory concentration (MIC) of *S. pneumoniae* D39 before and after the initial time-kill curve experiment.**

Values represent mean ± SD (n = 3). The MIC value was determined for one randomly selected surviving clone of the initial time-kill assay. MIC values before and after the initial time-kill experiment did not significantly differ (Student's T-test), except for the MIC for moxifloxacin in the diluted stationary growth phase that was significantly lower for the repeated experiment (p = 0.001).

MIC (µg/mL)	EUCAST breaking points		D39	Clone after antibiotic treatment in diluted stationary phase	Clone after antibiotic treatment in exponential phase
	Sensitive (µg/mL)	Resistant (µg/mL)			
**Amoxicillin**	≤ 0.5	≥ 1	0.007 ± 0.002	0,009 ± 0,004	0,010 ± 0,002
**Cefuroxime**	≤ 0.25	> 0.5	0.022 ± 0.005	0,031 ± 0,003	0,034 ± 0,018
**Moxifloxacin**	≤ 0.5	≥ 0.5	0.233 ± 0.006	**0,071 ± 0,039**	0,282 ± 0,075
**Vancomycin**	≤ 2	≥ 2	0.450 ± 0.111	0,426 ± 0,080	0,588 ± 0,155

Table S2: **The biphasic model describes the time-resolved killing data better than the uniphasic model.** Mathematical analyses of the entire dataset, with a global model containing a condition-dependent structure, and of the individual conditions by comparing the fitting of two non-linear fixed-effect models (uniphasic versus biphasic model) to time-kill curves. Probability that the model is correct is determined using the Akaike's information criterion (AIC), the Bayesian information Criterion (BIC) and the Log-Likelihood (LogLik). **For the model where the AIC/BIC is lower and the LogLik is higher, the probability is higher that that model is correct.** Dil stat, diluted stationary growth phase; Exp, exponential growth phase.

	AIC	BIC	LogLik	AIC	BIC	LogLik
**Global model comparison (across all treatments)**						
Uniphasic	915.8	951.2	-448.9			
Biphasic	481.4	579.8	-215.7			
**Amoxicillin**	**Exp**			**Dil stat**		
Uniphasic	204.1	208.5	-100.1	36.3	38.9	-16.2
Biphasic	115.7	124.6	-53.9	33.0	38.2	-12.5
**Cefuroxime**	**Exp**			**Dil stat**		
Uniphasic	146.2	150.7	-71.1	28.9	**31.5**	-12.4
Biphasic	104.6	113.5	-48.3	**28.8**	34.0	**-10.4**
**Moxifloxacin**	**Exp**			**Dil stat**		
Uniphasic	219.0	223.4	-107.5	25.0	27.6	-10.5
Biphasic	88.8	97.7	-40.4	18.3	23.5	-5.2
**Vancomycin**	**Exp**			**Dil stat**		
Uniphasic	118.4	122.9	-57.2	-7.2	**-4.6**	5.6
Biphasic	68.7	77.6	-30.4	**-8.2**	-3.0	**8.1**

Table S3: **Mathematical analysis of the fitting of a biphasic non-linear fixed-effect model to kill curves of amoxicillin (amox), cefuroxime (cef), moxifloxacin (mox) and vancomycin (van) against *S. pneumoniae* D39.** 95% confidence intervals of the parameters are given between brackets. P_0, persister fraction at the start of treatment; K_n , killing rate of normal cells; K_P, killing rate of persister cells; Exp, exponential growth phase; Dil stat, diluted stationary growth phase.

Antibiotic	Growth phase	P_0	K_n	K_p
**Amox**	Exp	0.0012<br>(-0.0098 – 0.0034)	1.3240<br>(1.1281 – 1.5200)	0.2628<br>(0.1703 – 0.3553)
	Dil stat	0.2431<br>(-0.1645 – 0.6507)	2.5802<br>(-1.3650 – 6.5254)	0.5586<br>(0.2708 – 0.8463)
**Cef**	Exp	0.0050<br>(-0.0117 – 0.0216)	0.9259<br>(0.7873 – 1.0644)	0.3166<br>(0.1566 – 0.4765)
	Dil stat	0.4650<br>(-0.0644 – 0.9944)	3.7800<br>(-10.5042 – 18.0642)	0.5067<br>(0.2981 – 0.7153)
**Mox**	Exp	0.0040<br>(0.0014 – 0.0065)	2.4595<br>(2.0477 – 2.8713)	0.3611<br>(0.3187 – 0.4035)
	Dil stat	0.1374<br>(-0.1644 – 0.4391)	1.7510<br>(0.5172 – 2.9848)	0.5756<br>(0.2320 – 0.9192)
**Van**	Exp	0.0002<br>(-0.0005 – 0.0009)	0.8854<br>(0.8194 – 0.9513)	0.2510<br>(0.0962 – 0.4058)
	Dil stat	0.6008<br>(0.1265 – 1.0750)	2.0722<br>(-3.5989 – 7.7432)	0.2706<br>(0.1353 – 0.4059)

Table S4: **Minimum inhibitory concentration (MIC) of reference strains and clinical isolates.** All strains are sensitive to amoxicillin (amox), cefuroxime (cef), moxifloxacin (mox) and vancomycin (van) according to the EUCAST breaking points, except for strain 85 that displays resistance towards cefuroxime and for CI 7 that displays resistance towards moxifloxacin. Values represent mean ± SD (n = 3).

Strain	Amox	Cef	Mox	Van	Strain	Amox	Cef	Mox	Van
**D39**	0,007 ± 0,002	0,022 ± 0,005	0,233 ± 0,006	0,450 ± 0,111	**CI 3**	0.014 ± 0.008	0.019 ± 0.006	0.073 ± 0.026	0.348 ± 0.101
**TIGR4**	0.006 ± 0.002	0.032 ± 0.013	0.347 ± 0.069	0.424 ± 0.092	**CI 4**	0.010 ± 0.003	0.018 ± 0.007	0.118 ± 0.006	0.365 ± 0.087
**ATCC49619**	0.036 ± 0.008	0.239 ± 0.145	0.242 ± 0.004	0.245 ± 0.002	**CI 5**	0.012 ± 0.004	0.017 ± 0.008	0.376 ± 0.096	0.535 ± 0.072
**R6**	0.011 ± 0.002	0.016 ± 0.007	0.226 ± 0.061	0.216 ± 0.017	**CI 6**	0.014 ± 0.001	0.015 ± 0.000	0.255 ± 0.010	0.496 ± 0.006
**85**	0.359 ± 0.105	**5.215 ± 0.895**	0.094 ± 0.026	0.301 ± 0.084	**CI 7**	0.010 ± 0.004	0.012 ± 0.003	**0.637 ± 0.236**	0.297 ± 0.106
**88**	0.009 ± 0.004	0.083 ± 0.020	0.146 ± 0.027	0.246 ± 0.024	**CI 8**	0.011 ± 0.004	0.013 ± 0.002	0.311 ± 0.048	0.567 ± 0.097
**CI 1**	0.014 ± 0.001	0.030 ± 0.000	0.459 ± 0.131	0.401 ± 0.033	**CI 9**	0.011 ± 0.002	0.022 ± 0.006	0.200 ± 0.005	0.517 ± 0.037
**CI 2**	0.124 ± 0.017	0.115 ± 0.007	0.084 ± 0.028	0.487 ± 0.007	**CI 10**	0.013 ± 0.003	0.012 ± 0.004	0.145 ± 0.030	0.644 ± 0.190

Table S5: **Primers used for plasmid construction and validation of the *spxB* and *lytA* knockout mutants.**

Primer	Sequence
**Construction of pLytA**	
For_lytA_first500	5’-TCCCGTTGAATATGGCTCATCCATTTAGCAAGATATGGATAAGGGTCAAC-3’
Rev_lytA_first500	5‘- TATGGTCGACCTGCAGGCGGCCGCGAATTCACTAGTGATTATGGAAATTAATGTGAGTAAATTAAGAACAGATTTGCCTCAAGT -3’
For_kan	5‘- ATCCATATCTTGCTAAATGGATGAGCCATATTCAACGGGAAACG -3’
Rev_kan	5‘- TTCTCAATATCATGCTTAAATTAGAAAAACTCATCGAGCATCAAATGAAACT -3’
For_lytA_last500	5‘- GCATGCTCCCGGCCGCCATGGCGGCCGCGGGAATTCGATTTTATTTTACTGTAATCAAGCCATCTGGCTCTACT- 3’
Rev_lytA_last500	5‘- TGCTCGATGAGTTTTTCTAATTTAAGCATGATATTGAGAACGGCTTGAC -3’
**Construction of pSpxB**	
For_spxB_first500	5‘- TATGGTCGACCTGCAGGCGGCCGCGAATTCACTAGTGATTATGACTCAAGGGAAAATTACTGCATCTG -3’
Rev_ spxB_first500	5‘- GCGATCACCGCTTCCCTCATGAAGTTTACTGGAATTTCAACAACAGCTGG -3’
For_strep	5‘- CGATCTGGATTGTCTTTCTTTTATTTGCCGACTACCTTGGTGATCT -3’
Rev_strep	5‘- TTGAAATTCCAGTAAACTTCATGAGGGAAGCGGTGATCGCC -3’
For_ spxB _last500	5‘- CCAAGGTAGTCGGCAAATAAAAGAAAGACAATCCAGATCGCCAAG -3’
Rev_ spxB _last500	5‘- GCATGCTCCCGGCCGCCATGGCGGCCGCGGGAATTCGATTTTATTTAATTGCGCGTGATTGCAATCCTTCTTCTTCCA -3’
**cPCR to check integration**	
For_lytA_cPCR	5‘- TGCGCTGTTCTGATTTGAAAGA -3’
Rev_lytA_cPCR	5‘- AAAGGAGTTTCTGGTTCTGGAT -3’
For_spxB_cPCR	5‘- TATGGTCGACCTGCAGGCGGCCGCGAATTCACTAGTGATTATGACTCAAGGGAAAATTACTGCATCTG -3’
Rev_spxB_cPCR	5‘- GCATGCTCCCGGCCGCCATGGCGGCCGCGGGAATTCGATTTTATTTAATTGCGCGTGATTGCAATCCTTCTTCTTCCA -3’
**qPCR to check expression**	
For_lytA_qPCR	5‘- CAGATTTGCCTCAAGTCGGC -3’
Rev_lytA_qPCR	5‘- ATTCTGGGTCTTTCCGCCAG -3’
For_spxB_qPCR	5‘- TCTCCGCTCTTTGCGACAAT -3’
Rev_spxB_qPCR	5‘- TGTTGAATGCTCCATCACCCA -3’
For_gdh	5‘- GGAGACCTGGCTAAACGCAA -3’
Rev_gdh	5‘- GGTCTACGGGCAGTTCCAAT -3’
